# Prognostic Implications of Clinical, Laboratory and Echocardiographic Biomarkers in Patients with Acute Myocardial Infarction—Rationale and Design of the ‘‘CLEAR-AMI Study’’

**DOI:** 10.3390/jcm12175726

**Published:** 2023-09-02

**Authors:** Stylianos Daios, Vasileios Anastasiou, Dimitrios V. Moysidis, Matthaios Didagelos, Andreas S. Papazoglou, Nikolaos Stalikas, Thomas Zegkos, Efstratios Karagiannidis, Lemonia Skoura, Georgia Kaiafa, Kali Makedou, Antonios Ziakas, Christos Savopoulos, Vasileios Kamperidis

**Affiliations:** 1 First Department of Cardiology, AHEPA University Hospital, School of Medicine, Faculty of Health Sciences, Aristotle University of Thessaloniki, St. Kyriakidi 1, 54636 Thessaloniki, Greece; stylianoschrys.daios@gmail.com (S.D.); vasianas44@gmail.com (V.A.); dimoysidis@gmail.com (D.V.M.); manthosdid@yahoo.gr (M.D.); nstalik@gmail.com (N.S.); zegkosth@gmail.com (T.Z.); stratoskarag@gmail.com (E.K.); tonyziakas@hotmail.com (A.Z.); 2 Athens Naval Hospital, Dinokratous 70, 11521 Athens, Greece; anpapazoglou@yahoo.com; 3 Department of Microbiology, AHEPA University Hospital, School of Medicine, Faculty of Health Sciences, Aristotle University of Thessaloniki, St. Kyriakidi 1, 54636 Thessaloniki, Greece; mollyskoura@gmail.com; 4 First Propedeutic Department of Internal Medicine, AHEPA University Hospital of Thessaloniki, Aristotle University of Thessaloniki, 54636 Thessaloniki, Greece; gdkaiafa@yahoo.gr (G.K.); chrisavopoulos@gmail.com (C.S.); 5 Laboratory of Biochemistry, AHEPA General Hospital, Faculty of Health Sciences, School of Medicine, Aristotle University of Thessaloniki, St. Kyriakidi 1, 54636 Thessaloniki, Greece; kmakedou@auth.gr

**Keywords:** acute myocardial infarction, coronary artery disease, predictive biomarkers, echocardiography, personalized medicine

## Abstract

Background: Acute myocardial infarction (AMI) remains a major cause of death worldwide. Survivors of AMI are particularly at high risk for additional cardiovascular events. Consequently, a comprehensive approach to secondary prevention is necessary to mitigate the occurrence of downstream complications. This may be achieved through a multiparametric tailored risk stratification by incorporating clinical, laboratory and echocardiographic parameters. Methods: The ‘‘CLEAR-AMI Study’’ (ClinicalTrials.gov Identifier: NCT05791916) is a non-interventional, prospective study including consecutive patients with AMI without a known history of coronary artery disease. All patients satisfying these inclusion criteria are enrolled in the present study. The rationale of this study is to refine risk stratification by using clinical, laboratory and novel echocardiographic biomarkers. All the patients undergo a comprehensive transthoracic echocardiographic assessment, including strain and myocardial work analysis of the left and right heart chambers, within 48 h of admission after coronary angiography. Their laboratory profile focusing on systemic inflammation is captured during the first 24 h upon admission, and their demographic characteristics, past medical history, and therapeutic management are recorded. The angioplasty details are documented, the non-culprit coronary lesions are archived, and the SYNTAX score is employed to evaluate the complexity of coronary artery disease. A 24-month follow-up period will be recorded for all patients recruited. Conclusion: The ‘‘CLEAR-AMI” study is an ongoing prospective registry endeavoring to refine risk assessment in patients with AMI without a known history of coronary artery disease, by incorporating echocardiographic parameters, biochemical indices, and clinical and coronary characteristics in the acute phase of AMI.

## 1. Introduction

Despite ongoing advances in interventional management for patients with acute myocardial infarction (AMI), they continue to exhibit a substantial risk for cardiovascular complications [1]. Patients with AMI present at various clinical stages and suffer from myocardial damage and systemic inflammation to different extents. An advanced clinical stage indicates poorer prognosis in the acute phase and raises the risk for future cardiovascular complications, rehospitalization and death [2,3].

The inflammatory response in patients with AMI emerges shortly after reperfusion and peaks within the initial days following revascularization [4]. Inflammatory processes play a significant role in the pathophysiology of left ventricular (LV) remodeling and cardiac repair mechanisms, reflecting not only the extent of myocardial damage but also the vulnerability of individual lesions in patients with AMI [4,5,6].

Echocardiography is the mainstay imaging modality for evaluating the LV systolic function after revascularization in patients with AMI. LV ejection fraction (LVEF) is the most widely utilized index of systolic function in clinical practice [7]. However, it merely represents LV volumetric changes per cardiac cycle, which may not reflect accurately the post-AMI myocardial damage. Echocardiographic LV global longitudinal strain (GLS) has demonstrated superior predictive value to LVEF, in the short-term post AMI [8,9]. However, GLS does not account for LV dyssynchrony or LV afterload, which may be impaired after AMI. Myocardial work has been proposed as a novel echocardiographic parameter, which accounts for intrinsic myocardial function, dyssynchrony and the loading conditions [10,11].

The utilization of risk scores for early risk stratification and prognosis in patients with AMI is advocated in the current guidelines [2]. The Global Registry of Acute Coronary Events (GRACE) and Thrombolysis in Myocardial Infarction (TIMI) risk scores are employed to evaluate risk stratification post-AMI. Nevertheless, these scores primarily depend on clinical factors and standard biochemical indices without accounting for the LV myocardial damage and the inflammation burden [12]. Additionally, the Synergy Between Percutaneous Coronary Intervention with Taxus and Coronary Artery Bypass Graft Surgery (SYNTAX) score is merely based on angiographic features of dismal prognosis [2].

Therefore, this study aims to identify clinical, laboratory, echocardiographic and angiographic features that could refine available risk assessment schemes post-AMI. The ultimate goal is to develop a contemporary predictive risk model that includes clinical parameters, angiographic characteristics, biochemical biomarkers of systemic inflammation and echocardiographic myocardial work indices to identify high-risk patients in the acute phase and predict downstream cardiovascular complications.

## 2. Materials and Methods

### Study Design and Population

**Figure 1:**
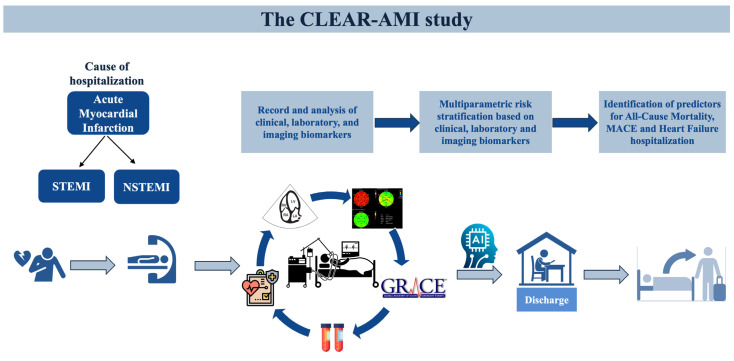
Study design diagram. Clinical, echocardiographic and laboratory features of 500 consecutive AMI patients without a history of coronary artery disease, will be evaluated to determine outcome predictors. Abbreviations: STEMI, ST-segment elevation myocardial infarction; NSTEMI, Non-ST-segment elevation myocardial infarction; MACE, Major Adverse Cardiovascular Event.

The ‘‘CLEAR-AMI’’ (ClinicalTrials.gov Identifier: NCT05791916) is a prospective, investigator-initiated, non-interventional study including patients with first AMI, ST-segment elevation myocardial infarction (STEMI) and Non-ST-segment elevation myocardial infarction (NSTEMI), hospitalized at the First Department of Cardiology of AHEPA University Hospital in Thessaloniki, Greece. All the patients undergo emergency coronary angiography in line with current guidelines [2]. The study adheres to the fundamental guidelines delineated in the Declaration of Helsinki [13] and the rules of good clinical practice, and has been approved by the Ethics Committee of the Aristotle University of Thessaloniki (reference number: 6.582/2022). The whole study design is presented in Figure 1.

This study aims to enroll a consecutive series of 500 patients presenting with first STEMI or NSTEMI and who are undergoing primary or emergency coronary angiography. A written informed consent is provided before enrollment for each eligible patient. Patients with a history of coronary artery disease, previous AMI or previous coronary intervention (percutaneous or surgical) will be excluded. Upon attaining the cohort of 500 patients, subgroup analyses will be conducted for NSTEMI and STEMI patients. The detailed eligibility criteria are described in Table 1.

## 3. Data Collection and Study Procedures

Demographic characteristics, baseline medical history, medication, prior diagnostic and therapeutic interventions, and clinical presentation of hospitalization will be recorded for all patients. In addition, patient laboratory data including complete blood count, biochemical, hormonal and coagulation mechanism control, high sensitivity troponin T levels (hs-cTNT) (admission and peak values) and N-terminal pro-B-type natriuretic peptide values will be collected within 24 h upon admission and during hospitalization. Moreover, the traditional lipid profile of the participants will be collected in addition to Lipoprotein A, and apolipoproteins B and A1. Special emphasis is placed on the assessment of inflammatory markers, including high-sensitivity C-reactive protein (hs-CRP), neutrophil-to-lymphocyte ratio (NLR), interleukin-6 (IL-6), soluble urokinase plasminogen activator receptor (suPAR), and admission glucose value, in order to evaluate stress-induced hyperglycemia. The prognostic impact of the investigated inflammatory markers (hs-CRP, IL-6, SuPAR) will be adjusted for potential cofounding variables such as concomitant autoimmune diseases and acute nosocomial infections as appropriate.

Clinical characteristics and risk scores, including Killip Class, GRACE risk score, TIMI-STEMI and ΤΙΜΙ-NSTEMI will also be recorded. Additionally, the coronary angiography of eligible patients will be assessed by two independent experienced interventional cardiologists who will be blinded to the demographic and clinical patient features. Angiographic data such as lesion characteristics, SYNTAX score and coronary dominance will also be taken into consideration.

All the eligible patients with AMI will be invited to visit the hospital one month post-AMI for a stress ECG test using the treadmill test. The test will be performed while the patient is on medication to assess the drugs’ effectiveness, their exercise capacity, and the chronotropic competence.

The primary endpoint will be all-cause mortality. The secondary endpoints will include (i) cardiovascular mortality (ii) heart failure rehospitalization and (iii) a composite of cardiovascular mortality, non-fatal acute myocardial infarction, unplanned PCI and heart failure hospitalization.

Artificial intelligence, specifically machine learning tools such as Extreme Gradient Boosting (XGBoost), will be used to identify clinical laboratory, echocardiographic and angiographic biomarkers with significant prognostic value and create potent diagnostic algorithms and predictive models. XGBoost stands as a prominently employed machine learning technique that is recognized for its exceptional efficacy and various functionalities, including regression, classification, and ranking quandaries [14]. It utilizes an iterative process of recursive binary partitioning to pinpoint the most advantageous division at each step, culminating in the development of an improved model [14].

All eligible patients with AMI will be actively encouraged to participate in a post-myocardial infarction exercise-based cardiac rehabilitation program as recommended by the current guidelines [2]. AMI patients will be encouraged to follow a personalized exercise program consisting of three sessions per week and supervised by a trainer under doctors’ directions. The aerobic and anaerobic exercises selected will be based on the age, gender, and physical status of each patient. The duration will initially be 30 min and the intensity will be low. Both the duration and intensity will be increased gradually according to the patients’ progress, aiming to exercise 5 times per week, 1 h per session with moderate intensity. The maximum heart rate obtained will be based on the stress ECG test that they will have in advance of the rehabilitation at 1 month post-AMI.

### 3.1. Echocardiographic Analysis

A detailed and thorough transthoracic echocardiographic evaluation will be conducted within 48 h of admission after undergoing coronary angiography. All transthoracic echocardiographic studies will be performed by certified operators using high quality equipment (Vivid E95 and Vivid S70, GE Healthcare, Chicago, IL, USA). Offline analysis with proprietary software will be performed using electrocardiogram-triggered echocardiographic data in a cine-loop format and analyzed by EchoPac software version 206 (GE Vingmed Ultrasound). In particular, strain analysis will be performed by Automated Functional Imaging software (GE Healthcare) via the EchoPac software version 206 (GE Vingmed Ultrasound). All the analyses will be conducted by two dedicated cardiologists with expertise in imaging, blinded to the participants’ clinical information. 

Cardiac chamber linear dimensions, volumetric quantification and Doppler analysis will be performed in line with current recommendations [15,16]. The LVEF will be calculated using Simpson’s biplane method. Diastolic function indices will be assessed as per contemporary recommended criteria [16]. Among others, indices of mitral inflow, annular tissue velocities, and the derived trans-mitral to averaged mitral annulus lateral and septal early diastolic velocity ratio (E/e’) will be evaluated. Indices of right ventricular (RV) performance, such as fractional area change (FAC), tricuspid annular plane systolic excursion (TAPSE), systolic movement of the RV lateral wall using tissue Doppler imaging (S’), and pulmonary artery systolic pressure (PASP), will be measured [17].

Longitudinal strain of the LV, RV and left atrium (LA) will be measured by two-dimensional speckle tracking echocardiography. To obtain images of the LV, the apical two-, three- and four-chamber views will be used. Images of the LA will be acquired via the apical two-chamber and four-chamber view. The apical four-chamber RV-focused view will be utilized for the RV. To determine the GLS, the peak systolic longitudinal strain of all segments will be averaged and calculated for each chamber. 

Myocardial work indices for the LV will be calculated non-invasively using a vendor-specific commercially available software package. To determine the parameters of myocardial work, peak systolic LV pressure and GLS of the LV will be incorporated into the formula outlined in a previous study [10]. Four distinct parameters of myocardial work will be computed: (a) LV global work index (mmHg %), which represents the overall work encompassed by the LV pressure–strain loops; (b) LV global constructive work (LVGCW, mmHg %), which is the work accomplished in systole during myocardial shortening as well as the lengthening of the myocardium in the period of isovolumic relaxation; (c) LV global wasted work (LVGWW, mmHg %), which reflects the work of inappropriate systolic myocardial lengthening and myocardial shortening in the isovolumic relaxation period; and (d) LV global work efficiency (%), denoting the proportion of appropriately utilized work by the LV myocardium, calculated using the equation: (LVGCW/[LVGCW + LVGWW]) × 100%.

The analysis of indices for myocardial work of the right ventricle will be conducted by modifying a proprietary software initially developed for assessing left ventricular myocardial work, as explained in a previous study [11]. Similar to the non-invasive approach for evaluating left ventricular myocardial work introduced by Russel et al., the software estimates force-segment length loops for the right ventricular myocardium using pressure–strain loops [10]. Instead of systolic and diastolic blood pressure, the software takes into account PASP and pulmonary artery diastolic pressure (PADP). Pulmonary artery mean pressure (PAMP) is derived using the following equation: the average gradient between the right ventricle and the right atrium plus the mean pressure of the right atrium [18]. The mean right ventricular–right atrial pressure is calculated by analyzing the tricuspid regurgitation velocity–time integral. PADP is estimated as PADP = 1.5 × [PAMP − (PASP/3)] [15,18].

The RV GLS, PASP, and PADP measurements are derived using of right heart valve events. This synchronization enables the generation of pressure–strain loops for the RV, which are derived non-invasively. The analysis of these loops allows for the derivation of four distinct parameters that assess RV function in a manner similar to that for the LV parameters mentioned above.

### 3.2. Statistical Analysis

The baseline clinical, echocardiographic, laboratory and angiographic features will be examined and compared by the chi-square test for categorical variables for each group. Student’s t-test, or the analysis of variance (ANOVA) will be used for continuous variables. When assumptions of normality are not met, non-parametric tests (Mann–Whitney U, Wilcoxon etc.) will be performed. Continuous variables will be represented by mean ± standard deviation (SD) or median (1st–3rd quartile). Categorical variables will be summarized by frequencies and percentages (%).

Univariable and multivariable logistic regression analyses, and Cox regression analyses will be applied to identify independent predictors at follow-up. Multivariate models will include only the co-variates that are significantly associated with the endpoint at univariate analysis. Receiver operating curves will evaluate the specificity and sensitivity of the resulting echocardiographic, clinical, angiographic and laboratory biomarkers for the prediction of early and late mortality, and rehospitalization. A likelihood ratio test will be used to assess the additional increase in the chi-square value in order to evaluate the potential incremental prognostic value of novel imaging or laboratory parameters over baseline models. To assess the clinical prognosis of several groups of patients with specific echocardiographic or laboratory characteristics, a time-to-event analysis will be performed according to the Kaplan–Meier method. A log-rank test will be used to compare the event rates. The significance threshold for all statistical tests will be considered the two-tailed *p* value of 0.05. The outcomes will be reported with 95% confidence intervals. Data management and statistical analyses will be conducted using SPSS software, version 26 (IBM SPSS Statistics) and R version 3.4.4 (R Foundation for Statistical Computing, Vienna, Austria). 

To calculate the sample size of the study, the G*Power software version 3.1.9.6 was used. It was estimated that to detect an odds ratio > 1.5, approximately 500 patients with AMI will be required with a power of 90%, a statistical significance level < 0.05 and assuming a dropout rate of 20%. The sample size calculations were based on previously published studies on the evaluation of myocardial work indices in patients with AMI [19,20].

## 4. Discussion and Expected Results

The ‘’CLEAR-AMI’’ study is an ongoing prospective cohort trial of patients hospitalized with first AMI. This study aims to provide a novel investigation of risk stratification with a special emphasis on echocardiographic LV myocardial damage and inflammation, and to further improve the available risk scores. Specifically, this study seeks—by combining clinical, laboratory, echocardiographic and angiographic parameters easily applicable in clinical practice—to profile subjects with a high risk of future events. To the best of our understanding, this is one of few prospective studies to comprehensively attempt a thorough, multidimensional risk stratification of patients suffering from AMI without a history of coronary artery disease.

As far as novel imaging biomarkers are concerned, only a limited number of studies have investigated the prognostic significance of myocardial work indices in patients with AMI. Lustosa et al. conducted a study involving 507 patients with STEMI and found that reduced global LV myocardial work efficiency (GLMWE) (<86%) was associated with an elevated risk of all-cause mortality compared with patients with preserved GLMWE (≥86%) [19]. Additionally, GLMWE demonstrated incremental prognostic value beyond LVEF and exhibited greater predictive strength compared with LV GLS when integrated into the prognostic model [19]. In another study by Butcher et al., the value of a different myocardial work parameter, global work index, was assessed in 197 individuals with STEMI. Lower global work index values (<750 mmHg%) were independently associated with an increased risk of all-cause mortality [20]. Notably, the global work index outperformed conventional echocardiographic parameters for assessing LV function, such as LVEF and LVGLS [20]. Taken together, these findings suggest that myocardial work parameters offer additional clinical value compared with the LVEF and LVGLS parameters commonly utilized in clinical practice. Lower values of myocardial work can serve as useful markers to stratify high-risk AMI patients who may benefit from aggressive titration of medical therapy and closer follow up [20].

Several studies have also addressed the prognostic role of RV dysfunction in patients with AMI with the use of non-speckle tracking echocardiographic parameters [17,21,22,23]. Despite its widespread applicability in heart failure [24,25], RV strain remains understudied in patients with AMI. RV free wall LS has emerged as a prognostic factor offering additional prognostic value beyond clinical and echocardiographic data [26]. RV dysfunction assessed by the conventional echocardiographic parameters of RV and RV free wall LS was an independent outcome predictor in an unselected group of 502 STEMI patients [27]. RV GLS has only been assessed in a cohort of 282 patients with inferior STEMI, where it emerged as an independent predictor of MACE, even after adjusting for multiple covariates [28]. RV myocardial work, which integrates the afterload through PASP and strain analysis for the evaluation of contractile function, represents an echocardiographic index that could offer additive information on RV performance post-AMI, with the potential to discern alterations in response to acute therapeutic interventions. The aim of this study is to broaden the existing research by exploring the short- and long-term prognostic role of RV myocardial work in patients with AMI.

The extent of inflammation, in the context of acute myocardial ischemia, is directly proportionate to infarct healing and the subsequent formation of scar tissue [29,30]. However, it is also well established that an exaggerated and persistent inflammatory response exerts detrimental effects on myocardial tissue, leading to exacerbated damage and ultimately contributing to worse clinical outcomes [31]. In AMI, the inflammatory response is accompanied by the secretion of various cytokines, including IL-6. IL-6, a cytokine of interest, exhibits pleiotropic effects, including protection of myocytes against oxidative stress. IL-6, along with hs-CRP and hs-cTNT, demonstrates an important prognostic role for adverse outcomes during the early phase following STEMI, and for MACE prediction during a long-term follow-up [32]. In a similar fashion, blood glucose has been independently associated with MACE prediction in STEMI patients characterized by stress-induced hyperglycemia (blood glucose on admission > 140 mg/dl), even in patients without a history of diabetes mellitus [33]. However, the association of inflammatory markers with novel echocardiographic parameters and their role in the assessment of the infarct and inflammatory burden, as well as in the prediction of MACE, are yet to be investigated.

In line with the hypothesis that there are a considerable number of pathways underlying atherosclerosis and AMI that remain to be discovered, this study aims to elaborate metabolomic and inflammatory biomarkers, such as the admission values of Lipoprotein A, Apolipoprotein A1 and B, suPAR and interleukin-6 (IL-6). The fundamental basis is that the metabolic and inflammatory characteristics of patients significantly contribute to the development of cardiovascular disease, and specifically coronary artery disease and AMI [33,34,35,36,37,38]. The prognostic roles and associations of these parameters will be assessed in this study. Furthermore, the integration of artificial intelligence techniques may facilitate the development of clinically significant polygenic risk scores [39,40], thereby offering potential advancements in risk prediction and patient management.

The utilization of scoring tools can also improve risk assessment post-AMI. Among the well-validated instruments, the GRACE (Global Registry of Acute Coronary Events) score stands out. The GRACE score incorporates eight clinical and biochemical variables and has recently been endorsed with a class IIa recommendation in the European guidelines to assess the risk of in-hospital mortality following AMI [2]. The GRACE score has demonstrated outstanding discriminatory capability for predicting the occurrence of in-hospital death [41]. In its latest iteration, the GRACE 2.0 score employs values obtained from regression models with nonlinear functions, specifically β coefficients, to derive a cumulative estimate of the likelihood of an unfavorable outcome. Notably, this updated approach eliminates the need for conversion into a point system [42]. Evidence suggests that the inclusion of inflammatory markers in the scoring system can provide valuable prognostic information [43] since the patients with AMI are usually characterized by an extensive myocardial inflammation, which triggers a systemic inflammatory response [44]. Elevated levels of inflammatory markers such as hs-CRP have also been associated with in-hospital cardiac events in AMI, independently of the GRACE risk score [45]. Interestingly, this traditional stratification score does not include echocardiographic parameters. Thus, the elaboration of novel imaging biomarkers could contribute further to the clinical profiling of patients with AMI and refine risk stratification.

It is advised for individuals with various clinical conditions to follow an exercise-centered cardiac rehabilitation program that serves as an efficacious strategy to foster a well-balanced way of living and manage the associated risk factors. In patients with AMI, this approach aims to improve all-cause and cardiovascular mortality and morbidity, concurrently augmenting health-related quality of life, as recommended by the current guidelines with a Class I indication, and Level of Evidence A [2,46]. A meta-analysis of 63 studies that included 14,486 patients revealed the beneficial effect of exercise training in the context of a cardiac rehabilitation program in patients with CAD, resulting in a reduction of up to 25% in cardiovascular mortality and a concomitant decrease of 18% in hospital readmissions [47]. In addition, substantial evidence demonstrates the positive influence of cardiac rehabilitation on left ventricular diastolic function, a parameter closely linked to exercise tolerance [48]. Thus, the implementation of a cardiac rehabilitation program in our study will significantly improve the outcome and the quality of life of our patients.

## 5. Limitations

Several limitations of the study should be acknowledged. Despite the prospective character of the study and the meticulous echocardiographic protocol that is followed, its single-centre nature will entail the need for future confirmation of the results from other centres to ensure their clinical validity. Future studies that include other ethnicities and races and investigate the influence of patients’ genetic profiles, aspects which are not examined in the current study, should be conducted to account for the intrinsic diversity across patient cohorts and verify the applicability and universality of our findings.

## 6. Conclusions

This prospective study of a real-world cohort of patients suffering from AMI aspires to shed light on the clinical, laboratory, angiographic and imaging biomarkers that are implicated in the prognosis of patients with AMI and to upgrade the existing risk assessment practices. Hence, individual profiling may eventually lead to the development of personalized risk-stratification models that could inform tailored therapeutic decisions. 

## Figures and Tables

**Table 1:** Inclusion and exclusion criteria of the “CLEAR-AMI Study”.

Inclusion Criteria	Exclusion Criteria
Age > 18 years Patients presenting with acute myocardial infarction (AMI) with or without ST segment elevation (based on the Fourth Universal Definition of Myocardial Infarction) Coronary angiography during hospitalization for AMI, in which at least one stenosis > 50% in a major epicardial coronary artery or a branch of at least 2 mm diameter	Not providing informed written consent Previous medical history of AMI Previous coronary revascularization Patients with a life expectancy < 1 year due to serious comorbidities Patients with limited echocardiographic acoustic window to perform analysis

## Data Availability

No new data were created or analyzed in this study. Data sharing is not applicable to this article.

## Abbreviations

AMI	acute myocardial infarction
NSTEMI	Non-ST-segment elevation myocardial infarction
STEMI	ST-segment elevation myocardial infarction
GLS	global longitudinal strain
GRACE	Global Registry of Acute Coronary Events
SYNTAX	Synergy Between Percutaneous Coronary Intervention with Taxus and Coronary Artery Bypass Graft Surgery
ΤΙΜΙ	Thrombolysis in Myocardial Infarction
LVGCW	left ventricle global constructive work
LVGWW	left ventricle global wasted work
LV	left ventricle
LVEF	left ventricular ejection fraction
RV	right ventricle
PADP	pulmonary artery diastolic pressure
PAMP	pulmonary artery mean pressure
PASP	pulmonary artery systolic pressure
MACE	Major Adverse Cardiovascular Event
